# Multiparametric MRI assessment of renal structure and function in acute kidney injury and renal recovery

**DOI:** 10.1093/ckj/sfaa221

**Published:** 2021-02-10

**Authors:** Charlotte Buchanan, Huda Mahmoud, Eleanor Cox, Rebecca Noble, Benjamin Prestwich, Isma Kasmi, Maarten W Taal, Susan Francis, Nicholas M Selby

**Affiliations:** 1 Sir Peter Mansfield Imaging Centre, School of Physics and Astronomy, University of Nottingham, Nottingham, UK; 2 Centre for Kidney Research and Innovation, University of Nottingham, Royal Derby Hospital Campus, Nottingham, UK; 3 National Institute for Health Research (NIHR), Nottingham Biomedical Research Centre, Nottingham, UK

**Keywords:** acute kidney injury, haemodynamic, multiparametric magnetic resonance imaging, oxygenation, renal function

## Abstract

**Background:**

Acute kidney injury (AKI) is associated with a marked increase in mortality as well as subsequent chronic kidney disease (CKD) and end-stage kidney disease. We performed multiparametric magnetic resonance imaging (MRI) with the aim of identifying potential non-invasive MRI markers of renal pathophysiology in AKI and during recovery.

**Methods:**

Nine participants underwent inpatient MRI scans at time of AKI; seven had follow-up scans at 3 months and 1 year following AKI. Multiparametric renal MRI assessed total kidney volume (TKV), renal perfusion using arterial spin labelling, T_1_ mapping and blood oxygen level-dependent (BOLD) R_2_* mapping.

**Results:**

Serum creatinine concentration had recovered to baseline levels at 1-year post-AKI in all participants. At the time of AKI, participants had increased TKV, increased cortex/medulla T_1_ and reduced cortical perfusion compared with the expected ranges in healthy volunteers and people with CKD. TKV and T_1_ values decreased over time after AKI and returned to expected values in most but not all patients by 1 year. Cortical perfusion improved to a lesser extent and remained below the expected range in the majority of patients by 1-year post-AKI. BOLD R_2_* data showed a non-significant trend to increase over time post-AKI.

**Conclusions:**

We observed a substantial increase in TKV and T_1_ during AKI and a marked decrease in cortical perfusion. Despite biochemical recovery at 1-year post-AKI, MRI measures indicated persisting abnormalities in some patients. We propose that such patients may be more likely to have further AKI episodes or progress to CKD and further longitudinal studies are required to investigate this.

## INTRODUCTION

Acute kidney injury (AKI) is a global healthcare concern, occurring in as many as 10–20% of people requiring hospital inpatient care [1]. AKI is associated with increased mortality, and is a risk factor for chronic kidney disease (CKD) and end-stage kidney disease [1]. A major limitation in current clinical practice is that serum creatinine, used to describe onset and stage of AKI, is a marker of renal excretory function that does not reliably inform the type or severity of renal injury, nor the subsequent cellular responses of damage and repair [2]. Furthermore, serum creatinine and estimated glomerular filtration rate (eGFR) are affected by changes in muscle mass, which occur commonly during hospitalization, and this can result in significant overestimation of renal recovery [3]. Renal imaging for AKI is currently restricted to ultrasound, largely performed to exclude urinary tract obstruction, and which often adds little to clinical decision-making. Improved methods of characterizing renal injury at the time of AKI and more accurately assessing degree of residual kidney damage are urgently required.

Multiparametric magnetic resonance imaging (MRI) techniques offer the potential to quantify pathophysiological processes relevant to AKI non-invasively, and a range of MRI measures such as longitudinal relaxation time (T_1_) mapping, arterial spin labelling (ASL) perfusion and total kidney volume (TKV) can be collected without the use of gadolinium contrast. By combining multiple MR measures within a single scan session a complete picture of the changes in kidney morphology, microstructure, haemodynamics and oxygenation can be provided. This enhances interpretation of MRI measures and allows better understanding of the pathophysiology of the changes as compared with collecting a single MRI measure alone. Multiparametric MRI has been applied in humans with CKD [4–6] and in renal transplantation [7], but to date, the few published clinical studies using MRI in AKI have used only a single MRI measure. Dong *et al.* [8] used renal ASL and showed cortical perfusion in AKI was significantly lower than in healthy volunteers (HVs). Prowle *et al.* [9] used phase-contrast MRI (PC-MRI) to demonstrate reduced renal artery blood flow in critically ill patients with AKI compared with HVs. Inoue *et al.* [10] used blood oxygen level-dependent (BOLD) MRI to measure R_2_* in AKI patients, but the results showed no consistent trends, while Bauer *et al.* [11] studied participants with acute tubular necrosis (ATN) and reported a wide range of R_2_* values and no significant change in R_2_* in response to furosemide.

MRI has been used in animal studies to study experimental AKI models. Hueper *et al.* [12] induced moderate and severe ischaemia–reperfusion injury (IRI) in a mouse model, and renal perfusion was measured using ASL at five timepoints between 1 and 28 days following the induction of AKI. Perfusion was decreased at Day 7 post-AKI for both the moderate and severe AKI model, and while this recovered by Day 21 for moderate injury, it did not recover following severe AKI. In a similar study by the same group, longitudinal relaxation time (T_1_), a marker of extracellular tissue fluid, increased following AKI, peaking at Day 7, and higher values were seen with more severe IRI [13]. Persistent elevations in T_1_ in the outer stripe of the outer medulla predicted subsequent kidney volume loss at Day 28.

Here, we report a pilot study using multiparametric renal MRI comprising TKV, T_1_ mapping, ASL perfusion and BOLD R_2_* mapping to assess changes in renal pathophysiology in participants with AKI and longitudinal follow-up of recovery over the subsequent year.

## MATERIALS AND METHODS

### Participants

Nine adults with AKI Stage 3 (defined using Kidney Disease: Improving Global Outcomes criteria) of >24 h duration were recruited from a renal inpatient setting (Royal Derby Hospital, UK). Participants were excluded if they had pre-existing CKD (eGFR <60 mL/min/1.73 m^2^ or renal transplant), AKI due to urinary obstruction, contraindications to MRI or if patients were deemed not medically stable for transfer to the MRI centre (Sir Peter Mansfield Imaging Centre, UK). The study was approved by the East Midlands Research Ethics committee and all participants gave written informed consent. The study was registered at ClinicalTrials.gov (NCT03578523).

### Participant assessments

Participants were studied at three timepoints: time of AKI (inpatient hospital stay as soon as possible after AKI onset), and as outpatients at 3 months and 1 year after the AKI episode. At each timepoint clinical data, measures of renal function and multiparametric renal MRI were obtained. Clinical data included demographics, medical history, medication, aetiology of AKI, blood pressure, height, weight and body mass index (BMI). Baseline serum creatinine was defined as the most recent stable serum creatinine prior to hospital admission. Protein-to-creatinine ratio (PCR) was measured from a single early morning urine sample (normal defined as <15 mg/mmol). eGFR was calculated from serum creatinine concentration (enzymatic colorimetric assay, Roche Diagnostics, Burgess Hill, UK) using the CKD Epidemiology Collaboration) formula [14], although eGFR was not calculated for inpatient measurements when renal function was not in steady state.

### Multiparametric renal MRI

#### Data acquisition

Multiparametric renal MRI was performed on a 3T Philips Ingenia scanner (Multi-Transmit, dStream), using the imaging protocols described previously in a study of HVs and CKD patients [4, 15]. The primary measures for this study were TKV, ASL perfusion, T_1_ mapping and BOLD R_2_*.

Balanced turbo field echo (bTFE) localizer scans (30 contiguous slices with 1.75 × 1.75 × 5 mm resolution collected in a single breath hold per orientation) were acquired in three orthogonal planes for quantification of kidney volume and planning of the five contiguous coronal oblique slices collected for multiparametric MRI. All multiparametric MRI data were acquired at end-expiration with a 288 × 288 mm^2^ field of view, 3 × 3 mm^2^ in-plane resolution and 5 mm slice thickness.

*ASL perfusion*. Respiratory-triggered flow alternating inversion recovery (IR) ASL data were acquired with in-plane pre- and post-saturation pulses immediately before and after the labelling pulses, and an inversion time (TI) of 1800 ms with selective (S)/non-selective (NS) thickness of 45/400 mm, and 25 S/NS pairs [15, 16] with a minimum repetition time (TR) of 4 s between ASL label and control images; if a subject had a respiratory cycle length shorter than this, then two respiratory cycles were waited for between the control/label measures. In addition, data were acquired across a range of TIs (four S/NS pairs at 300/500/700/900 ms) to assess inflow as well as an M_0_ scan for perfusion quantification. The M_0_ scan was collected with the same readout parameters as the ASL scan but without the ASL labelling and using a long TR to ensure full recovery of equilibrium magnetization. All data were collected with a spin-echo echo-planar-imaging (SE-EPI) readout (SENSE 2.3/TE 27 ms). The acquisition time for the ASL, inflow and M_0_ scans was ∼10 min, dependent on the subject's breathing rate.

*T_1_ mapping*. A respiratory-triggered IR T_1_ dataset (TIs: 200/300/400/500/600/700/800/900/1000/1100/1200/1300/1500 ms, temporal slice spacing 58 ms, minimum TR of 10 s) with fat-suppressed SE-EPI readout was collected [15] with matched geometry to the ASL data, to allow perfusion quantification. For improved corticomedullary differentiation (CMD), a single slice IR-bFFE (TI: 370/470/570/670/770/870/970/1070/1170/1270/1370/1470/1670 ms) T_1_ dataset with high in-plane resolution (1.5 × 1.5 mm^2^) was also acquired. The acquisition time for the T_1_ mapping scan was ∼3 min.

The ASL and T_1_ data acquisition both used a respiratory triggered scheme [15] to minimize respiratory-induced abdominal motion between images of differing contrast collected across the range of TIs. The respiratory trigger was applied at the peak of inspiration in the respiratory cycle and the image was then acquired at a constant time following this trigger, during the flat end-expiration period of the respiratory cycle by introducing a variable delay, Tv, between the respiratory trigger and the inversion pulse, which was followed by the TI, thus holding the time at which all image readouts are collected during end-expiration at a constant time of Tv + TI that was set to 1800 ms. The minimum TR of the T_1_ mapping scheme was set to 10 s to ensure full recovery between inversion pulses.

*BOLD data*. BOLD R_2_* data were acquired using a multi-echo fast field echo (mFFE) scheme (12 echoes, TE1/ΔTE = 5/3 ms, SENSE2, 25° flip angle). These data were collected in three 16 s breath holds (with two slices collected per breath hold).

#### Data analysis

Data analysis followed the techniques previously outlined in detail in Cox *et al.* [15]. TKV was computed by manually tracing the kidney on the coronal bTFE localizer images using Analyze9^®^ software (AnalyzeDirect, Overland Park, KS, USA) and corrected by body surface area (BSA). Multiparametric maps were collected in the same space for T_1_, perfusion and R_2_*, and maps were computed using in-house software (MATLAB, The Mathworks Inc., MA, USA). T_1_ maps were generated by fitting IR data to generate T_1_ and M_0_ maps for the SE-EPI: readout and an apparent T_1_ and M_0_ map for the bFFE readout. Perfusion-weighted images were computed by realignment and averaging of S/NS data sets, and subtraction of the averaged NS image from the S image, to create a single perfusion-weighted (ΔM) map. ΔM, inflow, M_0_ and T_1_ maps were then used in a kinetic model to calculate tissue perfusion maps [15]. The mFFE data were fit to form R_2_* maps by computing the log of the exponential signal decay.

Renal cortex and renal medulla masks were formed from the T_1_ maps, using a histogram of T_1_ values within both kidneys to define a T_1_ threshold for segmentation. Each mask was applied to each multiparametric map (T_1_, perfusion and R_2_*), generating a histogram of each multiparametric MRI measure in the cortex and medulla [15] to which a Gaussian curve was fit and the mode and full-width-at-half-maximum computed. Subsequently, the CMD in each measure was computed. Since no significant difference in multi-parametric measures was observed between right and left kidneys, the mean of both kidneys was used in analyses.

### Statistical analysis

A Shapiro–Wilk normality test was applied to clinical and MRI measures. Normal data are expressed as mean ± standard deviation (SD) and non-parametric data as median [interquartile range (IQR)].

#### Clinical data

Data were analysed using IBM SPSS version 25. Friedman and Wilcoxon signed-rank tests were used to test for significance.

#### MRI data

Narrative descriptions outline MRI measures across individual participants. For group statistical analysis, Prism 6 (GraphPad Software, Inc., La Jolla, CA, USA) was used. Significant differences between timepoints were assessed using a one-way analysis of variance (ANOVA) with Bonferroni correction for multiple comparisons, with P < 0.05 considered statistically significant and a subsequent *post hoc t*-test analysis between timepoints. Participants with missing data were excluded from the group analyses comparing timepoints.

## RESULTS

### Participant demographics

Nine participants with AKI underwent initial assessment and scanning, and seven attended both 3-month and 1-year follow-up assessments (two participants declined follow-up). Inpatient MRI scanning was performed at a median (IQR) of 6  (5) days after peak serum creatinine value. Participant characteristics are summarized in Table 1. All participants had AKI Stage 3 and two required acute renal replacement therapy, which was intermittent haemodialysis. Five of the nine AKI cases were associated with sepsis.

**Table 1.** Characteristics of study population

Number of patients	*N* = 9	*N* = 7
Age, years	46 ± 17	44 ± 18
Gender (male:female)	5:4	4:3
BMI, kg/m^2^	30.2 ± 6.1	30.9 ± 6.8
Baseline eGFR, mL/min/1.73 m^2^	91 ± 22 (range 68–133)	91 ± 23 (range 68–133)
Baseline serum creatinine, µmol/L	81 ± 23	82 ± 23
Admission serum creatinine, µmol/L	349 ± 201	330 ± 119
Peak serum creatinine, µmol/L	531 ± 250	449 ± 215
AKI Stage 3, %	9 (100)	7 (100)
Highest C-reactive protein during admission	78 (IQR 273)	78 (IQR 150)
Lowest systolic blood pressure during index admission, mmHg	111 ± 9 (range 100–125)	109 ± 9 (range 100–123)
Acute RRT, %	2 (22)	1 (14)
Comorbidity, %		
Diabetes mellitus	3 (30)	3 (43)
Hypertension	3 (30)	1 (14)
Cardiovascular disease	0	0
Aetiology of AKI, %		
Sepsis (%)	5 (56)	4 (57)
Hypovolaemia/hypoperfusion (%)	2 (22)	2 (29)
Tubulointerstitial nephritis (%)	1 (11)	1 (14)
Paracetamol overdose (%)	1 (11)	0
Medications prior to index hospital admission		
ACEi/ARB (%)	3 (33.3)	2 (29)
Diuretic (%)	1 (11)	1 (14)
CCB (%)	3 (33.3)	2 (29)
Average number of anti-hypertensive medications per patient, median (IQR)	0.7 (1.5)	0.6 (2)

Nine participants (*n* = 9) with AKI underwent initial assessment and scanning, and seven (*n* = 7) attended both 3-month and 1-year follow-up assessments (two participants declined follow-up). Normally distributed variables are presented as mean ± SD. Non-normally distributed variables are presented as median (IQR). Categorical variables are presented as a percentage (number). ACEi, angiotensin-converting enzyme inhibitor; ARB, angiotensin-receptor blocker; CCB, calcium channel blocker; RRT, renal replacement therapy.

### Clinical measures

**FIGURE 1:**
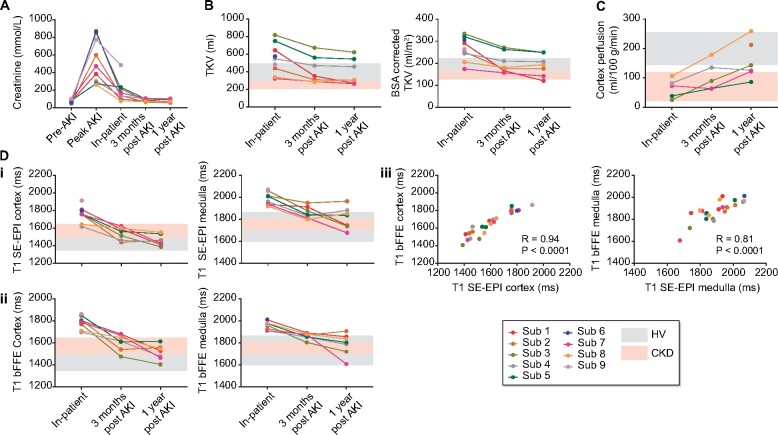
(**A**) Serum creatinine levels for each participant prior to AKI, at time of peak AKI, and at the time of each inpatient, 3-month post-AKI and 1-year post-AKI MRI scan. MRI measures collected at the three scan sessions—inpatient, 3-month post-AKI and 1-year post-AKI for (**B**) TKV and TKV corrected for BSA, (**C**) cortex perfusion and (**D**) T_1_ of the renal cortex and renal medulla for (i) the SE-EPI and (ii) bFFE image readout, and (iii) the correlation between bFFE and SE-EPI results. Grey and pink bands show the range of values in HV and CKD cohorts, respectively. Note, the range of renal cortex T_1_ in CKD [1D and 3D pink band] is elevated compared with the HV range [D and 3D grey band], whilst medulla T_1_ in CKD falls within the HV range.

Renal function had begun to recover in most patients at the first MRI scan; serum creatinine was significantly lower than at peak AKI but was significantly higher than baseline (pre-AKI). Table 2 summarizes clinical variables across the timepoints of the study for the seven participants who took part in all visits. During outpatient follow-up, renal function improved with all participants recovered to eGFR values of >60 mL/min/1.73 m^2^ at 3 months and 1 year, and corresponding serum creatinine values similar to baseline (pre-AKI) values (Figure 1A). Proteinuria was present in seven of the nine participants at the time of their inpatient MRI scan and while this did resolve in some patients, at 3 months three of seven patients displayed persistent proteinuria, and one patient had proteinuria (urine PCR 148 mg/mmol) at 1 year.

**Table 2.** Summary characteristics for the seven participants who attended all visits, i.e. completed baseline, 3-month and 1-year follow-up

Clinical measure	Baseline (pre-admission)	Peak AKI (highest creatinine measurement)	Time of first MRI scan (inpatient)	3-month post-AKI (outpatient)	1-year post-AKI (outpatient)
Serum creatinine, µmol/L	82 ± 23^a^	449 ± 215^a^	159 ± 61^a^	85 ± 17^a^	81 ± 16^a^
eGFR, mL/min/1.73 m^2^	91 ± 23	Not calculated		89 ± 22	99 ± 18
Systolic blood pressure, mmHg		109 ± 9	142 ± 21	128 ± 16	134 ± 15
C-reactive protein, mg/L		78 (150)		2 (8)	1.6 (5)
UPCR, mg/mmol		29 ± 49		18 ± 62	9 (134)
Recurrent AKI				0	0
Hospital readmission				0	0

Normally distributed variables are presented as mean ± SD. Non-normally distributed variables are presented as median (IQR).

a There was a significant change in serum creatinine over time (Friedman test), with a Wilcoxon signed rank test showing a significant difference between creatinine at peak AKI compared with all subsequent time measures (P < 0.01), creatinine at first MRI scan (inpatient) and creatinine at 3 months post-AKI (P = 0.004) and 1 year (P = 0.005). No significant differences were seen between creatinine measures at 3 months and 1 year post-AKI (P = 0.2). UPCR, urinary protein to creatinine ratio.

### MRI measures

#### Individual patient measures

Figure 1B–D shows each participant’s MRI measures of TKV, cortical perfusion and cortical and medullary T_1_ collected at the three timepoints. Reference ranges from comparator HV (61 ± 25 years, eGFR 92 ± 12 mL/min/1.73 m^2^) and CKD (61 ± 24 years, eGFR 39 ± 14 mL/min/1.73 m^2^) groups are also shown by Buchanan *et al.* [4]. Importantly, these data were collected using the same data acquisition measures and data analysis procedures as for the AKI data. Individual data are shown for all nine patients at time of AKI (Figure 1).

*TKV.* At the time of AKI, seven participants had TKVs higher than the HV range (396 ± 99 mL) (Figure 1B). TKV decreased at 3 months post-AKI in all participants and further decreased at 1 year post-AKI, although it remained above the range expected in HVs in two participants. Conversely, two patients had TKVs at 1-year that fell below that of HVs into the CKD range (322 ± 114 mL).

**FIGURE 2:**
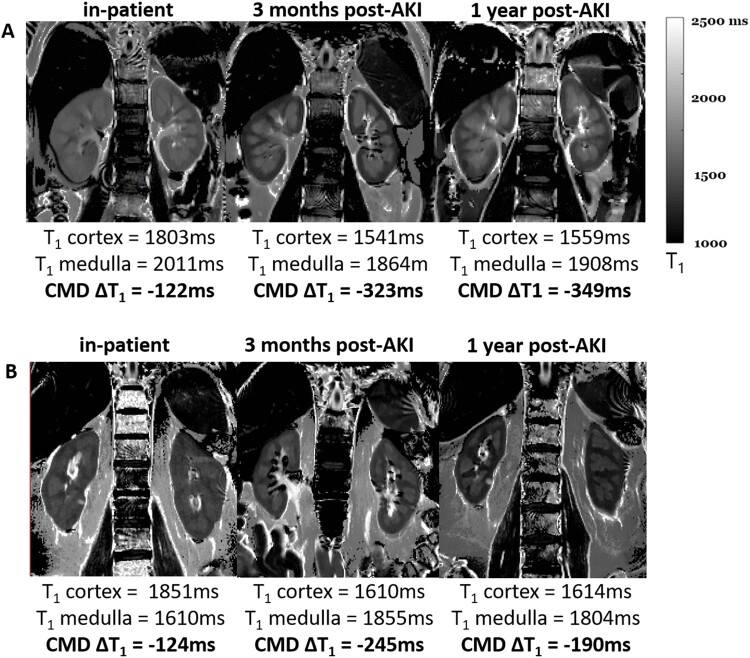
Example bFFE T_1_ maps through the long-axis of the kidney collected at the inpatient MRI scan and subsequently at 3 months and 1 year post-AKI. There is a clear lack of CMD for the inpatient T_1_ map, with T_1_ values of both the renal cortex and medulla being long. A reduction in the T_1_ of renal cortex and medulla is seen at 3 months and 1 year post-AKI, together with enhanced CMD. Data shown for (**A**) Subject 2 and (**B**) Subject 5, who had a significant drop in TKV.

*Longitudinal relaxation time, T_1_.* At time of AKI, cortical T_1_ values were higher than the HV range (1432 ± 87 ms) in all participants, and higher than the expected range in CKD (1574 ± 74 ms) in seven participants using the SE-EPI readout (Figure 1Di) and all participants using the bFFE readout (Figure 1Dii). Medullary T_1_ values at time of AKI were higher than the CKD range (1754 ± 50 ms) in all participants for both image readouts. Compared with the time of AKI, T_1_ values decreased at 3 months and further decreased at 1 year post-AKI, although values did not fall to the HV range in all participants. As expected, there was a strong correlation between individual SE-EPI and bFFE T_1_ measures for both renal cortex (*R* = 0.94, P < 0.001) and medulla (*R* = 0.81, P < 0.001). The higher in-plane resolution of the bFFE T_1_ maps provided improved delineation of changes in renal cortex and medulla (Figure 2); these images also illustrate the reduction of TKV from time of AKI to the 3-month and 1-year timepoints, particularly evident in Subject 5 (Figure 2B).

*Cortical perfusion.* Cortical perfusion as measured by ASL (*n* = 5) was lower than the HV range (200 ± 56 mL/100 g/min) at time of AKI; perfusion improved but remained low at 3 months post-AKI, returning to the HV range in only two participants at 1 year post-AKI (Figure 1C).

#### Group comparisons

**FIGURE 3:**
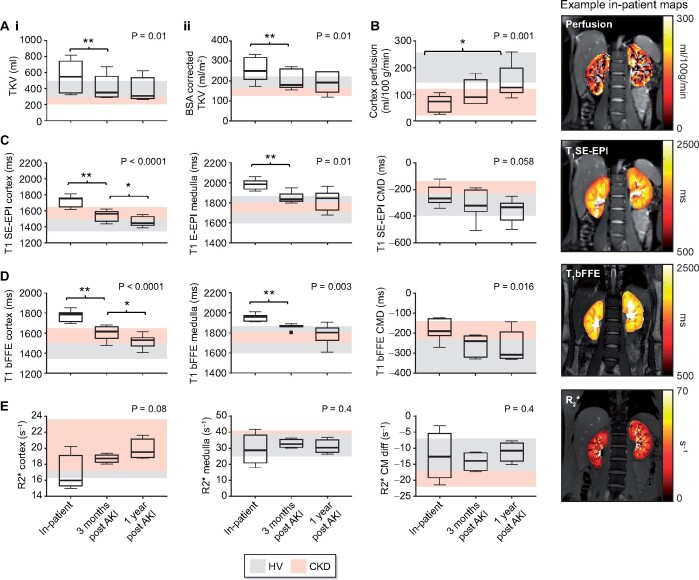
MRI results grouped across the seven participants who attended all visits. Data shown for (**A**) TKV [(i) TKV and (ii) BSA corrected TKV]; (**B**) cortex perfusion; (**C**) SE-EPI T_1_ values for the renal cortex, medulla and CMD; (**D**) bFFE T_1_ values for the renal cortex, medulla and CMD; (**E**) R_2_* measured in the renal cortex, medulla and CMD. P-values are shown for a repeated measures ANOVA (or Friedmann test for non-parametric data) across the three timepoints. *Post hoc t*-tests are shown as **P < 0.01 and *P < 0.05.

Figure 3 shows the MRI data grouped across the seven participants who attended all scan visits.

*TKV.* A significant reduction in both TKV (Figure 3Ai) and BSA corrected TKV (Figure 3Aii) was observed across time (inpatient: 553 ± 196 mL, 3 months: 421 ± 152 mL, 1 year: 397 ± 145 mL, P = 0.01), *post hoc* comparisons revealed a significant difference between inpatient and 3-month scan (P = 0.008). There was no significant difference between scans at 3 months and 1 year post-AKI (P = 0.1).

*Longitudinal relaxation time, T_1_.* A significant decrease in SE-EPI (Figure 3C) and bFFE (Figure 3D) T_1_ values was found over time (T_1_ cortex bFFE: inpatient: 1775 ± 55 ms, 3 months: 1606 ± 74 ms, 1 year: 1514 ± 69 ms, P < 0.001; T_1_ medulla bFFE: inpatient: 1954 ± 38 ms, 3 months: 1862 ± 29 ms, 1 year: 1789 ± 99 ms, P < 0.01). *Post hoc t*-tests showed differences between time of AKI and 3-month scan for both renal cortex and medulla (P = 0.001 for SE-EPI and P < 0.001 for bFFE). Between 3 months and 1 year post-AKI there was a significant reduction in cortical T_1_ (P = 0.047 for SE-EPI and P = 0.024 for bFFE); however, no significant difference was found in the medulla for either readout, with a wider range of T_1_ values. The CMD significantly increased (became more negative) over time for the bFFE T_1_ measure (P = 0.02), although this trend was not significant (P = 0.058) for the SE-EPI T_1_ maps, likely due to the reduced in-plane spatial resolution of the SE-EPI data to define the cortex and medulla.

*Cortical perfusion.* Cortical perfusion increased significantly across time (inpatient: 65 ± 33 mL/100 g/min, 3 months: 106 ± 50 mL/100 g/min, 1 year: 147 ± 66 mL/100 g/min, P = 0.001, Figure 3B), with a significant increase in perfusion between time of AKI and 1-year follow-up scan (P = 0.02), but no significant difference between time of AKI and 3-month scan, or 3-month and 1-year scan (P = 0.06 for each). Acceptable quality BOLD R_2_* data were only achieved in four patients due to motion artefacts during the breath-hold scan. Subjects for whom data quality was acceptable are shown in Figure 3E; BOLD R_2_* data showed a trend to increase over time, but this did not reach significance.

## DISCUSSION

In this study, we have performed multiparametric MRI in nine patients with AKI to non-invasively quantify longitudinal changes in renal pathophysiology. In all participants, serum creatinine recovered to baseline levels. At time of AKI, MRI data showed increased TKV, increased cortex/medulla T_1_ and reduced cortical perfusion compared with the expected ranges in HVs and people with CKD. TKV and T_1_ values decreased over time after AKI and returned to expected values in most but not all patients by 1 year. Cortical perfusion improved to a lesser extent and remained below the expected range in the majority of patients by 1 year post-AKI.

Clinically, there is a need to develop new methods to better assess the nature and severity of renal injury across the wide spectrum of clinical scenarios and patient groups in which AKI develops. Furthermore, chronic impairment of renal function after AKI often goes undetected due to the insensitivity of eGFR, as well as lack of testing and follow-up. Considerable efforts have been directed towards the development of plasma and urine biomarkers for earlier diagnosis compared with serum creatinine, or to assess risk of progression or lack of recovery, although few are in widespread clinical use [17]. Quantitative MRI provides ‘imaging biomarkers’ sensitive to pathophysiological processes occurring in AKI, including changes in tissue microstructure, cortical perfusion and oxygenation [18–20]. This combination of measures may address important clinical challenges, for example, identifying mechanisms of renal injury and assessment of AKI severity, measuring the time-course of resolution of AKI-induced abnormalities, differentiating between those who recover completely versus those who are left with chronic damage, and improving prognostic assessment of those at risk of clinically significant CKD progression. To date, the use of quantitative MRI to study AKI has been limited [8–11]; a multiparametric approach has not previously been attempted, nor has MRI been used to assess the recovery phase of AKI. Here, we used multiparametric MRI to study the human renal cortex and medulla at the time of AKI, and to describe changes during recovery.

We observed that TKV at the time of AKI was markedly increased compared with the HV range. Clinically, ultrasound-measured kidney size (reported as kidney length) is used to distinguish AKI from the diagnosis of CKD, which is associated with small kidneys. Less commonly, ultrasound can identify increased kidney size in some AKI such as infiltrative diseases, renal vein thrombosis and acute glomerulonephritis [21], but these causes of AKI were not present in our study. The observed increase in TKV was coupled with large increases in renal cortex and medulla T_1_, notably higher than for CKD in which T_1_ is elevated compared with HVs [4]. T_1_ measures were consistent across SE-EPI and bFFE measures, with the higher in-plane spatial resolution bFFE scan providing superior visual definition of cortex and medulla (to achieve as high spatial resolution for the SE-EPI acquisition, the echo time would become limiting). The pronounced increase in medulla and cortex T_1_ at the inpatient AKI scan is suggestive of oedema and/or inflammation of the renal parenchyma, which may also explain why TKV increased [22].

During recovery, TKV and T_1_ measures declined, consistent with resolution of oedema or inflammation from the acute phase of AKI. In most participants, TKV returned to the HV range by 1 year, although TKV remained elevated in two participants. Conversely, in two other participants, TKV fell to a value below the CKD range. This finding may suggest chronic, subclinical renal damage, consistent with animal studies associated with kidney volume loss proportionate to AKI severity and chronic damage [13]. However, this cannot be concluded with certainty as TKV was not measured in our patients prior to the AKI episode. Furthermore, some participants had persistent elevations in cortex and medullary T_1_ after 1 year, which may again reflect chronic, subclinical damage.

We observed reduced cortical perfusion at the time of AKI in comparison with the HV range. Reduced perfusion is regarded as a key initiating process in many forms of AKI (e.g. volume depletion, reduced cardiac output, systemic vasodilation in sepsis or renal vasoconstriction in hepato-renal syndrome) [23]. Our observation of reduced cortical perfusion in participants with predominantly sepsis and hypovolaemia-associated AKI is in agreement with previous human and animal studies using ASL. Dong *et al.* [8] used renal ASL to study 13 participants at the time of AKI and showed that renal perfusion in this group was significantly lower (223–392 mL/100 g/min) than for HVs (357–426 mL/100 g/min). Hueper *et al.* [12] showed a decrease in perfusion in a mouse model of IRI, with greater reductions seen in severe AKI that persisted to 28 days. Prowle *et al.* [9] used PC-MRI to demonstrate a significant reduction in renal artery blood flow [median 244 (165–662)  mL/min/m^2^] in 10 adults with established septic AKI compared with HVs (525 mL/min/m^2^), a magnitude of reduction in blood flow consistent with our findings for renal perfusion. In our study, perfusion increased post-AKI but remained in the CKD range at 1 year post-AKI in some participants. We speculate this represents vascular rarefaction and/or nephron loss and may indicate those participants more likely to have a further AKI episode or progress to CKD.

The shortened BOLD R_2_* is likely a result of decreased transverse relaxation rate (R_2_) due to inflammation, as shown in AKI in mice [24]. Inoue *et al.* [10] assessed BOLD in 23 AKI Stages 1–3 patients in comparison with HVs and CKD patients, but their results showed no consistent trends, which they attributed to the range of interstitial oedema and renal hypoxia in AKI. Bauer *et al.* [11] used BOLD R_2_* to study nine participants with ATN-induced AKI, assessed at baseline and after administration of furosemide. A wide range of R_2_* values was found for the AKI group and no significant change in R_2_* in response to furosemide.

In our analysis, MRI measures in AKI participants were compared with a CKD and HV group. The AKI participants age range was lower (46 ± 17 years), although overlapping with the HV and CKD comparator groups (61 ± 25 years). Any age dependence could result in the reference range HV and CKD perfusion values being lower and T_1_ values being higher [25], but this effect is likely to be minor and would result in more conservative estimates of difference.

Collection of PC-MRI (and a non-contrast-enhanced MR angiogram for planning) and diffusion-weighted imaging multiparametric scans were secondary measures included towards the end of the scan session, but these data were not collected or had to be discarded due to poor quality. There were challenges associated with scanning AKI participants, especially during their inpatient stay, due to the need to lie supine for a long period and perform breath-hold scans. In future, the use of recently available compressed sensing for body MRI [26], allowing data to be acquired in shorter scan periods and significantly shorter breath-hold lengths, will be explored. In addition, the use of a respiratory navigator rather than the respiratory bellows for respiratory gating will be trialled, as patients found the latter to be uncomfortable, limiting acquisition of the perfusion data at the inpatient scan. Furthermore, having evaluated the optimal measures for use in research studies, we envisage a smaller subset of the key measures would be used for future clinical application. A further limitation of this study is the small participant number due to the challenges associated with recruiting patients during acute illness. This resulted in some bias in favour of patients with less severe illness, as patients requiring intensive care management were not studied.

In conclusion, we have shown changes in multiparametric renal MRI measures of kidney volume, T_1_ and perfusion outside the healthy range at time of AKI. These MRI measures improved over the 1 year follow-up but did not return to the HV range in all participants, despite all achieving biochemical recovery. These results provide essential pilot data to inform the design of future larger studies.

## FUNDING

This work was supported by Animal Free Research UK and the Medical Research Council (grant number MR/R02264X/1). Animal Free Research UK is a UK medical research charity that funds and promotes non-animal techniques to replace animal experiments.

## AUTHORS’ CONTRIBUTIONS

S.F., N.M.S. and M.W.T. designed the study; C.B., H.M., R.N., I.K. and S.F carried out experiments; C.B., E.C., B.P. and S.F analysed the MRI data; H.M and R.N analysed the clinical data; S.F., N.M.S., M.W.T. and C.B. drafted the paper; all authors approved the final version of the manuscript.

## CONFLICT OF INTEREST STATEMENT

None declared. The results presented in this article have not been published previously in whole or part, except in abstract format.

## DATA AVAILABILITY STATEMENT

The data underlying this article will be shared on reasonable request to the corresponding author.

## References

[1] Hoste EAJ , Kellum JA, Selby NM et al Global epidemiology and outcomes of acute kidney injury. Nat Rev Nephrol 2018; 14: 607–625 30135570 10.1038/s41581-018-0052-0

[2] Selby NM. A comment on the diagnosis and definition of acute kidney injury. Nephron 2019; 141: 203–206 30661075 10.1159/000496441

[3] Prowle JR , Kolic I, Purdell-Lewis J et al Serum creatinine changes associated with critical illness and detection of persistent renal dysfunction after AKI. Clin J Am Soc Nephrol 2014; 9: 1015–1023 24742481 10.2215/CJN.11141113 PMC4046736

[4] Buchanan CE , Mahmoud H, Cox EF et al Quantitative assessment of renal structural and functional changes in chronic kidney disease using multi-parametric magnetic resonance imaging. Nephrol Dial Transplant 2020; 35: 955–910 31257440 10.1093/ndt/gfz129 PMC7282828

[5] Yu YM , Ni QQ, Wang ZJ et al Multiparametric functional magnetic resonance imaging for evaluating renal allograft injury. Korean J Radiol 2019; 20: 894 31132815 10.3348/kjr.2018.0540 PMC6536799

[6] Berchtold L , Friedli I, Crowe LA et al Validation of the corticomedullary difference in magnetic resonance imaging-derived apparent diffusion coefficient for kidney fibrosis detection: a cross-sectional study. Nephrol Dial Transplant 2020; 35: 937–945 30608554 10.1093/ndt/gfy389

[7] Friedli I , Crowe LA, Berchtold L et al New magnetic resonance imaging index for renal fibrosis assessment: a comparison between diffusion-weighted imaging and T1 mapping with histological validation. Sci Rep 2016; 6: 1–15 27439482 10.1038/srep30088 PMC4954968

[8] Dong J , Yang L, Su T et al Quantitative assessment of acute kidney injury by noninvasive arterial spin labeling perfusion MRI: a pilot study. Sci China Life Sci 2013; 56: 745–750 23740361 10.1007/s11427-013-4503-3

[9] Prowle JR , Molan MP, Hornsey E et al Measurement of renal blood flow by phase-contrast magnetic resonance imaging during septic acute kidney injury: a pilot investigation. Critic Care Med 2012; 40: 1768–1776 10.1097/CCM.0b013e318246bd85 22487999

[10] Inoue T , Kozawa E, Okada H et al Noninvasive evaluation of kidney hypoxia and fibrosis using magnetic resonance imaging. J Am Soc Nephrol 2011; 22: 1429–1434 21757771 10.1681/ASN.2010111143 PMC3148697

[11] Bauer F , Wald J, Bauer FJ et al Detection of acute tubular necrosis using blood oxygenation level-dependent (BOLD) MRI. Kidney Blood Press Res 2017; 42: 1078–1089 29197870 10.1159/000485600

[12] Hueper K , Gutberlet M, Rong S et al Acute kidney injury: arterial spin labeling to monitor renal perfusion impairment in mice-comparison with histopathologic results and renal function. Radiology 2014; 270: 117–124 24023073 10.1148/radiol.13130367

[13] Hueper K , Peperhove M, Rong S et al T1-mapping for assessment of ischemia-induced acute kidney injury and prediction of chronic kidney disease in mice. Eur Radiol 2014; 24: 2252–2260 24996794 10.1007/s00330-014-3250-6

[14] Levey AS , Stevens LA, Schmid CH et al.; for the CKD-EPI (Chronic Kidney Disease Epidemiology Collaboration). A new equation to estimate glomerular filtration rate. Ann Intern Med 2009; 150: 604–612 19414839 10.7326/0003-4819-150-9-200905050-00006 PMC2763564

[15] Cox EF , Buchanan CE, Bradley CR et al Multiparametric renal magnetic resonance imaging: validation, interventions, and alterations in chronic kidney disease. Front Physiol 2017; 8: ii4–ii14 10.3389/fphys.2017.00696 PMC5603702 28959212

[16] Gardener AG , Francis ST. Multislice perfusion of the kidneys using parallel imaging: image acquisition and analysis strategies. Magn Reson Med 2010; 63: 1627–1636 20512866 10.1002/mrm.22387

[17] Srisawat N , Kellum JA. The role of biomarkers in acute kidney injury. Critic Care Clin 2020; 36: 125–140 10.1016/j.ccc.2019.08.010 31733675

[18] Wolf M , de Boer A, Sharma K et al Magnetic resonance imaging T1- and T2-mapping to assess renal structure and function: a systematic review and statement paper. Nephrol Dial Transplantat 2018; 33: ii41–ii50 10.1093/ndt/gfy198 PMC6106643 30137583

[19] Pruijm M , Mendichovszky IA, Liss P et al Renal blood oxygenation level-dependent magnetic resonance imaging to measure renal tissue oxygenation: a statement paper and systematic review. Nephrol Dial Transplant 2018; 33: ii22–ii28 30137579 10.1093/ndt/gfy243 PMC6106642

[20] Odudu A , Nery F, Harteveld AA et al Arterial spin labelling MRI to measure renal perfusion: a systematic review and statement paper. Nephrol Dial Transplant 2018; 33: ii15–ii21 30137581 10.1093/ndt/gfy180 PMC6106644

[21] Faubel S , Patel NU, Lockhart ME et al Renal relevant radiology: use of ultrasonography in patients with AKI. Clin J Am Soc Nephrol 2014; 9: 382–394 24235286 10.2215/CJN.04840513 PMC3913238

[22] Rabb H , Griffin MD, McKay DB et al Inflammation in AKI: current understanding, key questions, and knowledge gaps. J Am Soc Nephrol 2016; 27: 371–379 26561643 10.1681/ASN.2015030261 PMC4731128

[23] Sharfuddin AA , Weisbord SD, Palevsky P et al Chapter 31: acute kidney injury. Brenner & Rector’s The Kidney, 10th edn, Vol. 3. Amsterdam, The Netherlands: Elsevier, 2016, 958–1011

[24] Hueper K , Rong S, Gutberlet ÞM et al T2 relaxation time and apparent diffusion coefficient for noninvasive assessment of renal pathology after acute kidney injury in mice comparison with histopathology. Invest Radiol 2013; 48: 834–842 23907103 10.1097/RLI.0b013e31829d0414

[25] Breidthardt T , Cox EF, Squire I et al The pathophysiology of the chronic cardiorenal syndrome: a magnetic resonance imaging study. Eur Radiol 2015; 25: 1684–1691 25577519 10.1007/s00330-014-3571-5

[26] Feng L , Benkert T, Block KT et al Compressed sensing for body MRI. J Magn Reson Imaging 2017; 45: 966–987 27981664 10.1002/jmri.25547 PMC5352490

